# Comparing standard screening questionnaires of canine behavior for assessment of cognitive dysfunction

**DOI:** 10.3389/fvets.2024.1374511

**Published:** 2024-05-21

**Authors:** Julia Haake, Sebastian Meller, Nina Meyerhoff, Friederike Twele, Marios Charalambous, Steven R. Talbot, Holger A. Volk

**Affiliations:** ^1^Department of Small Animal Medicine & Surgery, University of Veterinary Medicine Hannover, Hannover, Germany; ^2^Institute for Laboratory Animal Science and Central Animal Laboratory, Hannover Medical School, Hannover, Germany; ^3^Center for Systems Neuroscience Hannover, Hannover, Germany

**Keywords:** cognition, canine dementia, geriatrics, questionnaire, assessment tools

## Abstract

**Background:**

Canine cognitive dysfunction (CCD) is a common, yet underdiagnosed neurodegenerative disease affecting older dogs. Treatment is most effective when started early, so identifying mild cognitive decline in the earlier stages of the disease is considered important.

**Hypothesis/objective:**

To compare the results of three different standard screening questionnaires [Canine Dementia Scale (CADES), Canine Cognitive Assessment Scale (CCAS), and Canine Cognitive Dysfunction Rating Scale (CCDR)] for CCD diagnosis. Trainability, pain sensitivity, and fear were additionally assessed with the Canine Behavioral Assessment and Research Questionnaire (C-BARQ) in order to evaluate associations between the three dementia scales and behavior.

**Methods:**

An online survey containing all the mentioned questionnaires was designed for and distributed among owners of elderly dogs.

**Results:**

Data from 597 dogs were analyzed. Overall, the scores of the three CCD questionnaires correlated well with each other, especially those of the CADES and CCAS. The CADES was more sensitive in identifying dogs with already mild to moderate cognitive impairment, while the others classified them as still undergoing normal aging. CCD scores increased for all questionnaires with age with spatial orientation being a key feature in CCD development. Trainability assessed with the C-BARQ decreased significantly with severity of CCD signs, while pain sensitivity increased. Fear and anxiety was pronounced in animals with mild but not with severe CCD. These associations based on the C-BARQ were more clearly observable in relation to CADES and CCDR than CCAS.

**Conclusion/clinical relevance:**

The choice of screening questionnaire impacts the evaluation of cognitive status and severity of CCD. Thresholds for severity classification differ significantly and may have an impact on reliable assessment. Further longitudinal studies are required to determine which of the questionnaires investigated in this study is best suited for early detection of CCD.

## 1 Introduction

Canine cognitive dysfunction (CCD) is a highly prevalent neurodegenerative disease among the elderly dog population (1–3). Metabolic changes in the aging brain, such as glucose hypometabolism and mitochondrial dysfunction, (4–7), and neuropathological findings, like the accumulation of ß42-amyloid plaques (8–13), and cerebral atrophy (14) may be associated with cognitive decline leading to typical clinical signs that can be classified into different cognitive domains under the DISHA-A acronym: Disorientation, altered interaction, changes in sleep–wake-cycles, house-soiling, altered activity and anxiety (15, 16).

Even though these behavioral changes can negatively impact the dog’s quality of life (8) and caring for an affected dog is perceived as a burden by their owners (17), CCD is an underdiagnosed condition (1–3, 18). While research studies reveal a prevalence ranging from 14.2 to 68%, depending on the age group and study (1–3, 18), veterinary surgeons might not make the diagnosis in first opinion practice. One study reports that only 1.9% of dogs over the age of 8 years are formally diagnosed with CCD by a veterinarian (18).

A potential reason for this might be the absence of a definitive diagnostic path for CCD at the moment, making the diagnosis of the disease challenging (10). CCD is currently a diagnosis of exclusion and getting a detailed medical history and performing a general, orthopaedic, and neurological exam is crucial. This is because underlying chronic diseases, such as endocrinopathies, neurological conditions, pain, dental, gastrointestinal or urogenital diseases can mimic and aggravate the clinical signs of CCD (19–21).

Screening questionnaires are helpful to assess patients’ behavior and cognitive function and many different owner questionnaires have been designed to help diagnose CCD (8, 11, 22–27). They are currently the most important diagnostic tools (28) and have been shown to detect cognitive impairment accurately, especially in cases of severe impairment (28) and when regularly repeated. Their scores have been shown to correlate well with other diagnostic findings such as the accumulation of ß42-amyloid plaques (11, 24, 29). However, some scales are meant to be filled out by specialists (26) and these scores may differ from questionnaires completed by owners.

In addition, blood tests can be performed to rule out other diseases. Ideally, magnetic resonance imaging (MRI) and the examination of cerebrospinal fluid (CSF) should be conducted. Findings in dogs with CCD may include brain atrophy, ventricular enlargement, and leukoaraiosis (30). However, owners rarely wish to perform an MRI due to the risk of general anesthesia in geriatric pets, the high cost, and the lack of benefits for concurrent treatment (31). Furthermore, behavioral testing, like the Delayed Non-Matching to Position Test or the Practical Cognitive Test, has been used to detect cognitive impairment (32) in laboratory settings (27, 33, 34), but the use in a clinical setting is not established so far (10). More recent studies suggest that combining owner-based screening questionnaires with cognitive testing and assessment of concentration of the biomarker plasma neurofilament light-chain (pNfL) may help to reliably detect CCD in a clinical setting as well (35, 36).

Early CCD diagnosis is important. If drug and nutritional intervention is commenced early on, clinical signs of CCD may be improved and further degeneration can be delayed (8). Potential treatment options are different medications, such as selegiline or propentofylline (8, 37), and dietary modifications like medium-chain triglycerides (38–40), antioxidants (41–44) and certain nutrient blends (3, 34, 45–47), which are used to ameliorate some of these age-related behavioral problems and can improve signs of at least one cognitive domain.

Having a diagnostic tool, which reliably detects these subtle behavioral changes before the disease progresses drastically, is crucial for commencing an effective treatment. Some studies investigating the correlation between different screening questionnaires have been published (11, 28) and found that scores correlated well within the group of severely impaired dogs. However, as new screening questionnaires have been developed with the goal of early detection of CCD, it is still unclear which questionnaire is best suited for the detection of cognitive impairment and what influence the choice of questionnaire has on the final diagnosis.

Therefore, we planned to investigate the relationship between the three dementia questionnaires Canine Dementia Scale (CADES), Canine Cognitive Assessment Scale (CCAS), and Canine Cognitive Dysfunction Rating Scale (CCDR). In addition, the Canine Behavioral Assessment and Research Questionnaire (C-BARQ) was used, which was designed to assess general behavior problems in dogs and is used frequently in veterinary practice (48). The C-BARQ was not created to assess dementia. However, the perspective from a behavioral standpoint might provide interesting insights by overlaying the outcomes from the dementia scales and the behavioral scale. This might ultimately reflect whether the scores of the dementia scales are associated with behavior aspects sampled in ways other than through the dementia tools themselves, possibly revealing a certain grade of sensitivity of the dementia scales towards some domains of the prominently used behavior scale. The aim of the study was to determine the relations between the questionnaires and investigate differences in structure, behavior assessment, and cut-off values that may lead to different evaluations of cognitive status. The three different dementia scales were chosen because of their differing design and their varying levels of complexity. The results of this study will provide important information to keep in mind when diagnosing patients with CCD.

## 2 Materials and methods

An online questionnaire was created and hosted via LimeSurvey^®^ from November 2022 to February 2023. Owner consent regarding privacy policies was gained at the beginning of the survey. Owners of elderly dogs (≥8 years old) were recruited via Facebook and Instagram regardless of whether their dog had been diagnosed with CCD by a veterinarian or not.

General information about the patients’ owner, the dogs’ signalment, and medical history were gathered. Then, owners were asked to complete the questions of three screening questionnaires for CCD: The CADES, the CCAS, and the CCDR, which were not modified in our survey. In addition, behavioral domains of the C-BARQ associated with trainability, fear, and pain sensitivity were included for evaluation. Other behavior domains of the C-BARQ, such as “chasing” and “excitability,” were excluded as they seemed less relevant to the diagnosis of CCD. Owners were masked to the origin of the questions.

The Madari questionnaire (CADES) (26) was designed based on a study including 215 dogs between the age of 8 and 16.5 years. The patients were clinically examined by veterinarians, and radiography, ultrasound, electrocardiography examinations, urine, and blood analyses were performed to exclude underlying conditions that could affect their behavior. The CADES is based on previously designed questionnaires (3, 25) and consists of 17 items that can be assigned to four domains: “spatial orientation,” “social interaction,” “sleep–wake-cycles,” and “house-soiling.” The total score ranges from 0–95 points and classifies dogs into four groups: Normal aging and mild, moderate, or severe cognitive impairment. The score ranges per domain can be found in the Supplementary Table S1.

The Le Brech questionnaire (CCAS) (27) was designed from a study including 100 dogs. It was adapted from different existing questionnaires and includes 17 items from six different domains: “disorientation,” “social interaction,” “anxiety,” “activity level,” “sleep–wake-cycles,” and “learning and memory.” The total score ranges from 0–69 and classifies dogs into three categories: normal aging, mild/moderate, and severe cognitive impairment (Supplementary Table S2).

Lastly, the Salvin questionnaire (CCDR) (25) was created from data gathered from an online questionnaire that was completed by 957 owners of dogs aged 8 years or older. The dogs were not examined by a veterinarian before or after the completion of the questionnaire. The CCDR consists of 13 questions, seven of which examine the current frequency of behavioral entities distinguishing dogs with or without cognitive impairment, and six questions pertain to changes in frequency of specific behavioral entities over the last six months. The total score ranges from 13–80 points and classifies dogs into three groups: No signs of CCD, at risk for developing CCD, and displaying signs of CCD.

As the CCDR has been found to detect severe cognitive impairment reliably, we chose to compare this questionnaire with the CADES, which was designed to detect mild impairment as well. We also chose to compare these two validated diagnostic tools with the CCAS, which, much like the DISHA-A questionnaire, has not been validated but is used frequently in clinical settings due to its simplicity. Furthermore, we chose these questionnaires because of their differing structures: The CADES and CCAS separate different behaviors into domains, whereas the CCDR does not, but focuses more on differences in behavior frequencies across time.

The C-BARQ (48) was created from a study including 1851 dogs. It was designed to assess general behavior traits of dogs and includes 68 questions from 11 different domains: “Stranger-directed aggression,” “owner-directed aggression,” “stranger-directed fear,” “nonsocial fear,” “dog-directed fear or aggression,” “separation-related behavior,” “attachment or attention-seeking behavior,” “trainability,” “chasing,” “excitability,” and “pain sensitivity.” The score of each question ranges from 0–4 and is then multiplied by a factor determined in the mentioned study. In our survey, we chose to focus on the five domains “dog-directed fear (or aggression),” “stranger-directed fear,” “nonsocial fear,” “trainability,” and “pain sensitivity,” as the others seem to play a less important role in CCD-associated behavior.

Data were organized and analyzed using Microsoft Excel, Numbers (Apple Inc.), R, and GraphPad Prism 10. Descriptive and analytical statistical analyses were performed. Kruskal–Wallis tests with Dunn’s multiple comparisons tests were used to compare the ages of dogs between the different severity categories within each scoring system. A linear mixed effects regression with Restricted Maximum Likelihood (REML) was used to model the influence of age and the three questionnaires (CADES, CCAS, CCDR) on CCD scores. The degrees of freedom were estimated using Satterthwaite’s method, i.e., comparing linear coefficients ( $\hat{\theta }$ ). Each CCD evaluation uniquely categorized severity classes (normal, mild/moderate/at risk, and severe/CCD) by thresholds, depending on the final score. The severity category was included in the model as a random effect to address the issue of individual intercepts and CCD-type specific variance. The random effect’s importance was tested in a likelihood ratio test, in which the full model was tested against the Null model, including only the random effect. Construct concordance analysis was performed with Fleiss’ Kappa as an index of interrater agreement between the three questionnaires. Pairwise comparisons were stored in a 3 by 3 matrix. Principal component analyses (PCAs) were performed to investigate multi-variate relationships between the three screening questionnaires, as well as the respective domain scores of the CADES and the CCAS. The latter was not possible for the CCDR, as it does not separate the behavioral entities into domains. Confirmatory factor analyses (CFAs) of the CADES and CCAS were performed to assess their validity by examining whether their domains reliably measure the underlying latent constructs. Factor loadings were standardized by outcome and standard deviation and were expressed as percentages. Lastly, radar plots were designed to illustrate the importance and development of CADES and CCAS domains along CCD progression, as well as the importance and development of C-BARQ domains across severity categories in each analyzed CCD questionnaire. Radar plots were supported by Kruskal–Wallis and Friedman tests with Dunn’s multiple comparisons tests that were used to compare the importance of questionnaire domains between and within the different severity categories as well as between scoring systems. All tests were two-tailed and a value of *p* ≤ 0.05 was considered significant.

## 3 Results

In total, 597 of 1,480 questionnaires provided complete answers and were appropriate for analysis. Most participating owners were females (91%), and 41% of owners were between the ages of 46 and 60. Most of them were from Germany (87%), the United States (5%), or the United Kingdom (2%). The overall median time participants had owned dogs was 20 years, ranging from 1 to 64 years.

The study cohort consisted mainly of female spayed (41%) and male neutered (36%) dogs. The majority of dogs were mixed breeds (29%), the most common pure-breed dogs were Labrador Retrievers (7%) and Golden Retrievers (4%). Median age of dogs was 12.5 years, ranging from 8 to 19.4 years.

### 3.1 Medical history

Out of the 597 participating dogs, 15% (*n* = 88) had already received a formal CCD diagnosis from a veterinarian; 85% of these dogs had been diagnosed by their primary care veterinarian, while 15% had seen a neurologist.

Two-thirds of the owners reported also that their dogs suffered from diseases other than CCD, such as diseases of the musculoskeletal system (44%), like osteoarthritis or spondylosis. Other frequently reported diseases were heart disease (13%), endocrinopathies (12%), and nephropathies (8%). However, 32% of the owners did not report other comorbidities than behavioral changes, including CCD. Of the participating owners, 43% felt that their dog was currently in pain.

Medication for brain health was given to 19% of dogs, while 25% of dogs were not on any medication at that time. “Other” medication was administered in 45% of dogs, with most owners reporting routine medications such as vaccinations and deworming treatment (43%) (Supplementary Table S3).

### 3.2 Comparison of CCD questionnaires

#### 3.2.1 Distribution of dogs across severity categories and questionnaires

For each questionnaire, the distribution of classification of dogs (*n* = 597) into different severity categories was displayed (Supplementary Figure S1). For the CADES, 29% of dogs showed no cognitive impairment, 35% showed mild, 21% moderate, and 15% severe signs of CCD (Supplementary Figure S1A). When all dogs were analyzed with the CCAS, the proportions were similar for dogs without (46.5%) and those with mild cognitive impairments (49%). However, only 4.5% of dogs displayed severe CCD signs (Supplementary Figure S1B). The CCDR questionnaire categorized most dogs as experiencing normal aging (67%), while 22% were at risk for developing CCD, and 11% exhibited signs of CCD (Supplementary Figure S1C).

In the subgroup of dogs previously diagnosed with CCD by a veterinarian (*n* = 88), the CADES detected mild cognitive impairment in 13% of dogs, moderate impairment in 34%, and severe impairment in 52% of dogs. One dog (1%) showed no signs of cognitive impairment but had received a CCD diagnosis in the past nonetheless (Supplementary Figure S2A). According to the CCAS, 3% of dogs had no signs of CCD, 73% had mild, and 24% had severe CCD signs (Supplementary Figure S2B). Interestingly, the CCDR found 28.5% to be aging normally, 36.5% to be at risk for developing CCD, and 35% to display signs of CCD (Supplementary Figure S2C).

**Figure 1:**
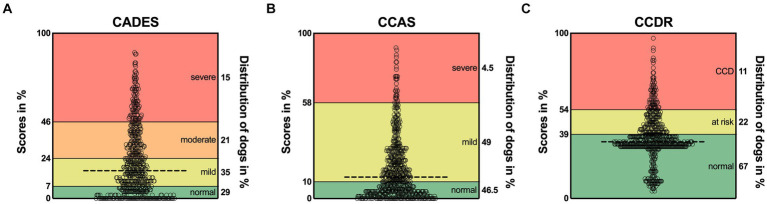
Total possible score ranges and dogs’ actual scores as percentage for **(A)** CADES, **(B)** CCAS, and **(C)** CCDR. The horizontal dashed line in each graph represents the corresponding median. Severity categories are color-coded, and their thresholds are displayed on the left *y*-axes as a percentage of the total possible score. The percentages on the right *y*-axes correspond to the distribution of animals in the respective categories. CCD, Canine cognitive dysfunction.

More precisely, an examination of the score ranges for each questionnaire revealed prominent differences in the severity classification thresholds (Figure 1), which potentially influenced the distribution of classified dogs across questionnaires. The CCDR exhibits a much larger range of normal aging compared to the CADES and CCAS, whereas the range for mild/moderate cases is broader in both latter ones. Accordingly, the overall median scores of dogs analyzed by the CADES and the CCAS fell within the range indicating mild cognitive impairment and of those analyzed by the CCDR in the range of normal aging. There was a large cluster of dogs visible in the CCDR at the score of around 35 (33% of the total score) and a smaller cluster with lower scores. All of these dogs were classified as aging normally (Figure 1C). A large number of dogs reached scores of zero for the CADES (13% of all dogs) and CCAS (11%).

#### 3.2.2 Evaluation of the effect of age on CCD severity across questionnaires

**Figure 2:**
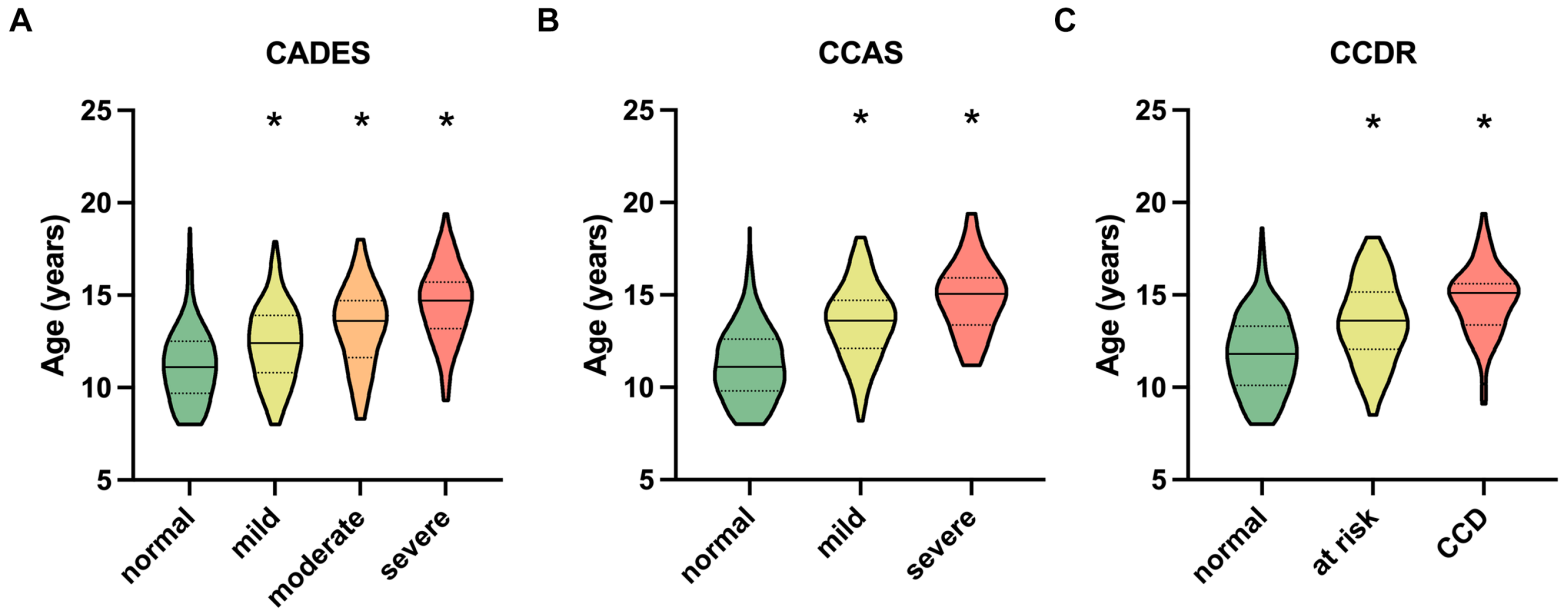
Truncated violin plots with median age (solid horizontal line), quartiles (dotted horizontal lines), and range of dogs’ ages in each severity category within each questionnaire. Kruskal–Wallis tests were significant for **(A)** the CADES, **(B)** the CCAS, and **(C)** the CCDR (*p* ≤ 0.0001). Dunn’s *post hoc* tests revealed significant differences in age between the categories (*p ≤ 0.0001; corresponding “normal” group as control). CCD, Canine cognitive dysfunction.

The respective ages per severity category for all questionnaires are shown in Figure 2. For the CADES, the median age in the normally aging group was 11.1 years (range 8–18.6) and 14.70 years (range 9.3–19.4) in the group of severely impaired dogs (Figure 2A). The distribution of age was similar for the CCAS and CCDR. The CCAS displayed median ages of 11.1 years (range 8–18.6) in the normal group and 15.1 years (range 11.2–19.4) in the severely impaired group (Figure 2B). For the CCDR, the median age in normal dogs was 11.7 years (range 8–18.6) and that in dogs with CCD was 15.1 years (range 9.1–19.4) (Figure 2C). The median age of dogs increased significantly with higher test scores and, therefore, severity category within the corresponding questionnaire (*p* ≤ 0.0001).

**Figure 3:**
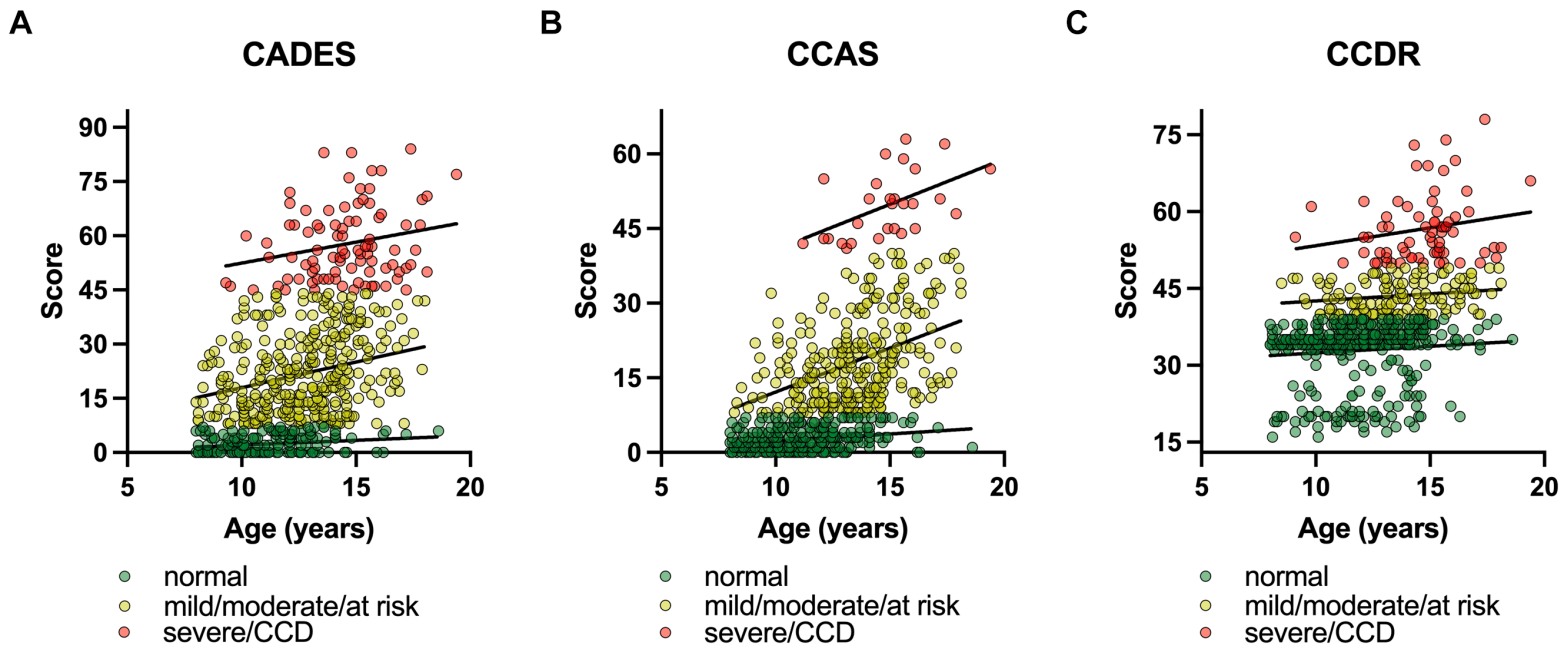
Canine cognitive dysfunction (CCD) scores as a function of age, facetted by questionnaire type, i.e., **(A)** CADES, **(B)** CCAS, and **(C)** CCDR. To ensure comparability between the questionnaires, severity categories were summarized as follows: (i) normal, (ii) mild/moderate/at risk, (iii) severe/CCD. In each CCD questionnaire, the severity levels were highlighted and fit with a linear function, representing the random intercepts term in the mixed model. The slopes of the functions between the groups differed significantly for **(A)** the CADES and **(B)** the CCAS, and **(C)** the CCDR over age. The interaction term of questionnaire type:age was significant in all three analyses (*p* ≤ 0.0001). Note the differences in intercepts between questionnaires and the variance in score ranges per severity categories.

The influence of age on the CCD scores was further assessed with a converged REML model with a severity class random effect. To ensure comparability between the questionnaires, severity categories were summarized as follows: normal, mild/moderate/at risk, severe/CCD (Figure 3). The model covered 85% of the model’s total variance (*σ*^2^_total_ = 375.01). The remaining residual variance (*σ*^2^_resl_ = 63.84) was, therefore, negligible. The importance of the random effect was further tested with a likelihood ratio test. The full model with the two fixed effects (age and CCD questionnaire) was tested against the Null model, containing only the random effect. The test showed a highly significant result for the severity variable in the model (*Χ*^2^ = 1989.9; *p* ≤ 0.0001).

In general, age had an impairing effect on canine cognitive function. Independent from the CCD questionnaire, a higher age resulted in significantly higher CCD values (Figure 3). Each additional year in age resulted in 0.89 (CI_95%_ [0.57; 0.85]) higher CCD scores ( $\hat{\theta }$ = 0.89, SE = 0.09, df = 1785.41, *p* ≤ 0.0001) on average. The model also found age-independent differences between questionnaires. CCAS reached the lowest average score with *μ*_CCAS_ = 10.22 (CI_95%_ [−15.32; 35.79]; $\hat{\theta }$ = −4.21, SE = 0.47, df = 1785.08, *p* ≤ 0.0001), followed by *μ*_CADES_ = 14.43 (CI_95%_ [−11.11; 39.99]; $\hat{\theta }$ = 14.43, SE = 11.25, df = 2.04, *p* = 0.326), which was also the intercept level in the linear model. The next higher questionnaire scores were reached by the CCDR with *μ*_CCDR_ = 36.55 (CI_95%_ [11.01; 62.12]; $\hat{\theta }$ = 22.12, SE = 0.49, df = 1785.13, *p* ≤ 0.0001). An additional analysis of variance with a type III error for estimating the degrees of freedom revealed a significant age:questionnaire interaction (*Χ*^2^ = 174.37, df = 2, *p* ≤ 0.0001), indicating that there were more complex relationships in terms of age and possibly severity classes that were not free of questionnaire-type dependent effects.

#### 3.2.3 Evaluation of agreement between CCD questionnaires

Construct concordance was used to analyze the agreement for the binary outcomes (“normal” or “not normal”) between the three scoring systems using Fleiss’ Kappa (Table 1). The binary outputs were generated for each animal based on different thresholds per CCD. Therefore, the binary response could vary. The analysis showed significant results (*n* = 597, *m* = 3, *z* = 18.4, *κ* = 0.436, *p* ≤ 0.0001). This result indicates moderate agreement (*κ* > 0.4) (49). The pairwise agreement analysis showed that CADES and CCAS shared the most similar evaluation results (*κ*_CADES:CCAS_ = 0.791).

**Table 1:** Pairwise interrater agreement of canine cognitive dysfunction raters (Fleiss’ Kappa values).

	CADES	CCAS	CCDR
CADES		0.791	0.603
CCAS	0.791		0.762
CCDR	0.603	0.762	

**Figure 4:**
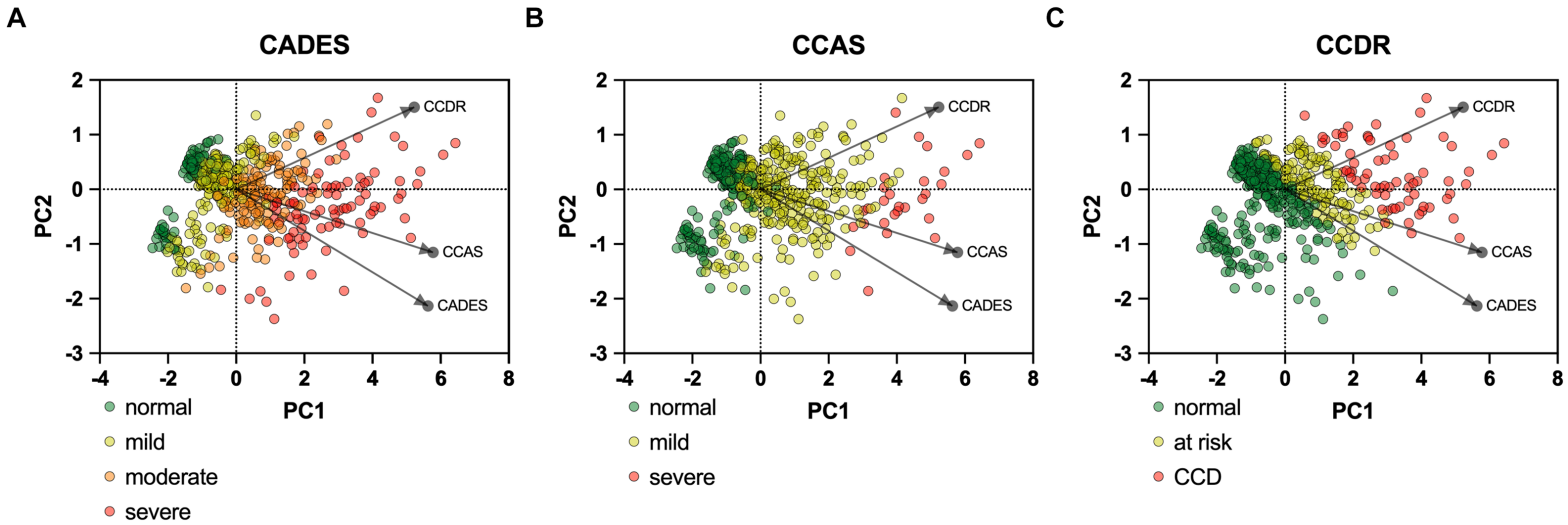
Principal component analysis (PCA) biplots of Canine cognitive dysfunction (CCD) scores and categorization in relation to the three questionnaires CADES, CCAS and CCDR. The colored data points (PCA scores) represent each dog and the general severity progression patterns (from green to red), whereas the black arrows and points represent the loadings for each questionnaire. The progression patterns and the loadings differ between all three scales, especially for both **(A)** CADES and **(B)** CCAS versus **(C)** CCDR, respectively.

PCAs of the CCD screening questionnaires also reflected the stronger relationship between CADES and CCAS, in contrast to the CCDR, and differences in the progression pattern and direction of the dogs, especially for CCDR versus CADES and CCAS (Figure 4).

#### 3.2.4 Evaluation of domains used in the CADES and CCAS

**Figure 5:**
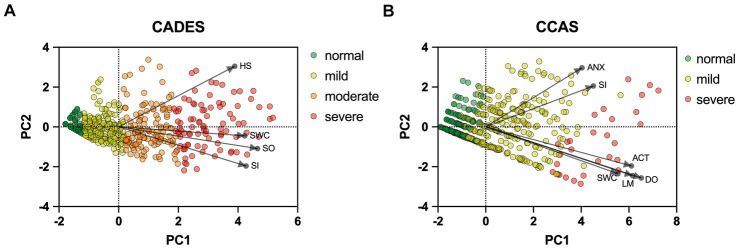
Principal component analysis (PCA) biplots of Canine cognitive dysfunction scores and categorization in relation to the **(A)** CADES and **(B)** CCAS domains “spatial orientation” (SO), “social interaction” (SI),” sleep–wake-cycles” (SWC), “house-soiling” (HS), “disorientation” (DO), “anxiety” (ANX), “activity level” (ACT), and “learning and memory” (LM). The colored data points (PCA scores) represent each dog and the general severity progression patterns (from green to red), whereas the black arrows and points represent the loadings for each domain. For the **(A)** CADES scoring, the domain of HS had less pronounced effects on the direction of progression patterns. For the **(B)** CCAS, the domains ANX and SI played a subordinate role for the main direction of CCD pattern progression. Please note that corresponding analyses were not conducted for the CCDR, as domains did not play a role in the evaluation there.

**Figure 6:**
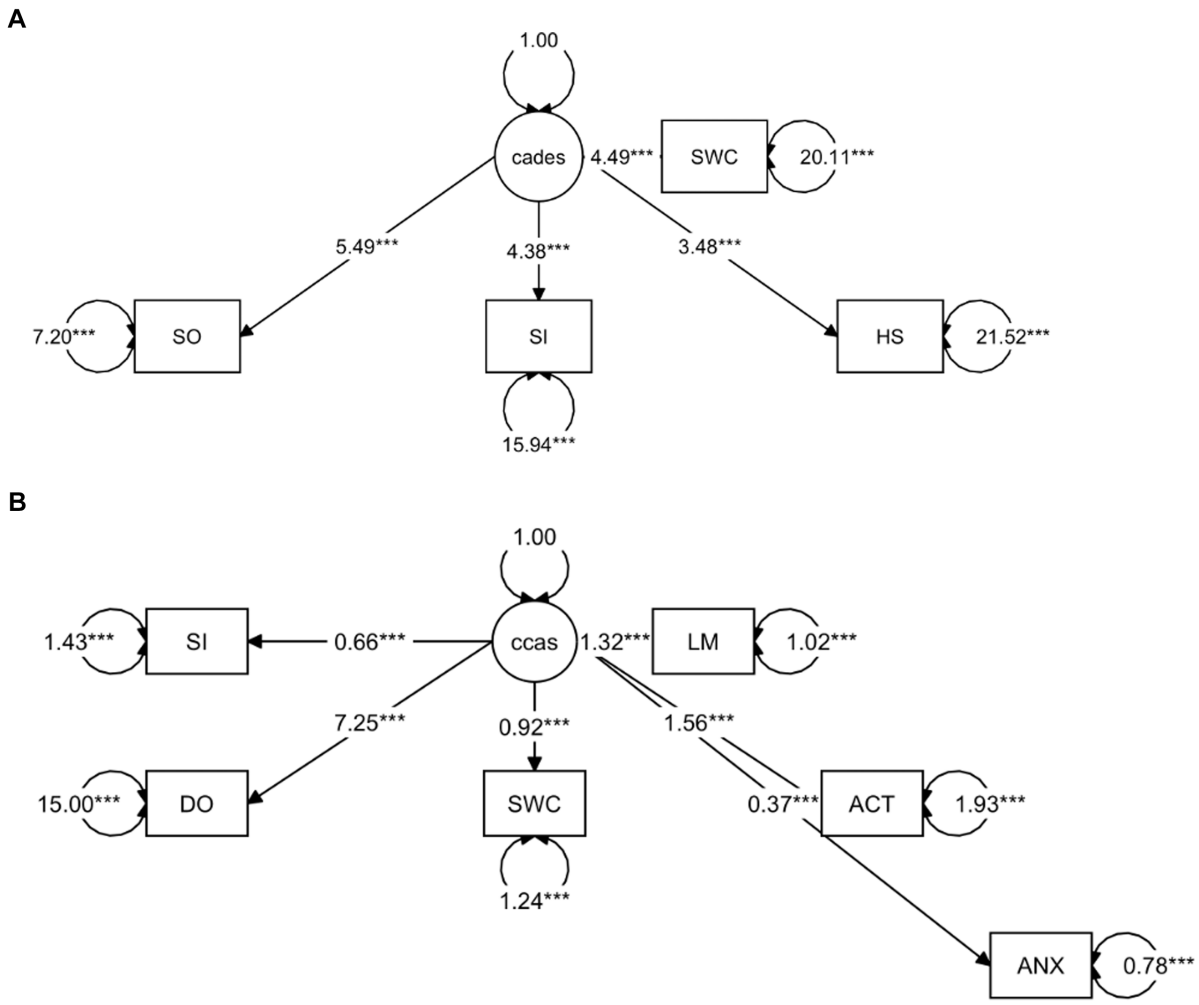
Confirmatory Factor Analysis of **(A)** CADES and **(B)** CCAS scoring in relation to their domains as indicators. The paths show the values of the factor loadings of the domains “spatial orientation” (SO), “social interaction” (SI), “sleep–wake-cycles” (SWC), “house-soiling” (HS), “disorientation” (DO), “anxiety” (ANX), “activity level” (ACT), and “learning and memory” (LM) on the latent variables CADES or CCAS. All domains loaded significantly on the total constructs (*p* ≤ 0.0001). The order of importance derived from the standardized factor loadings was as follows. For **(A)** CADES: SO (89.8%), SI (74%), SWC (70.8%), HS (60%); for **(B)** CCAS: DO (88.2%), LM (80%), ACT (74.6%), SWC (64%), SI (48.1%), ANX (38.9%). Values displayed on the arrowed circles represent estimates of the residual variances of each domain (*p* ≤ 0.0001). Please note that corresponding analyses were not conducted for the CCDR, as domains did not play a role in the evaluation there.

To provide a more specific view on the score systems, relationships between the CADES as well as CCAS domains and their severity categories were evaluated by using PCAs (Figure 5). In both scoring systems, the direction of the loadings (domains) explained well the direction of the progression patterns of CCD, which was not surprising since the severity outcome is composed by the summed-up scores of all domains. More specifically, the CADES domains “spatial orientation,” “social interaction,” and “sleep–wake-cycles” correlated well with each other and mainly impacted on PC1 and the main progression patterns while the “house-soiling” domain turned away from the other three domains and influenced PC1 and PC2 to a similar extent (Figure 5A). Additional CFA showed that, while all CADES domains loaded significantly on the total CADES score, the domain “spatial orientation” indicated the highest strength of relationship (standardized loadings) across items within the model (89.8%), and “house-soiling” the least (60%) (Figure 6A). Although the CFA Chi-square test was significant (*C*^2^ = 8.09, *p* = 0.017, *n* = 597), the Comparative Fit Index (CFI = 0.99) and the Tucker-Lewis Index (TLI = 0.98) indicated a good model fit.

The CCAS domains “disorientation,” “activity level,” “sleep–wake-cycles,” and “learning and memory,” correlated well with each other and had a strong influence, mainly on PC1 and the main progression patterns, whereas the “social interaction” and “anxiety” domains correlated well with each other and influenced PC1 and PC2 to a similar extent (Figure 5B). The CFA confirmed that the CCAS domains “anxiety” (38.9%) and “social interaction” (48.1%) showed the lowest standardized loadings on the total CCAS score, while the domains “sleep–wake-cycles” (64%), “activity level” (74.6%), “learning and memory” (80%), and “disorientation” (88.2%) showed the highest loadings on the total CCAS score (Figure 6B). Again, the CFA Chi-square test was significant (*C*^2^ = 70.32, *p* < 0.0001, *n* = 597). However, the Comparative Fit Index (CFI = 0.95) and the Tucker-Lewis Index (TLI = 0.92) still indicated an acceptable model fit.

**Figure 7:**
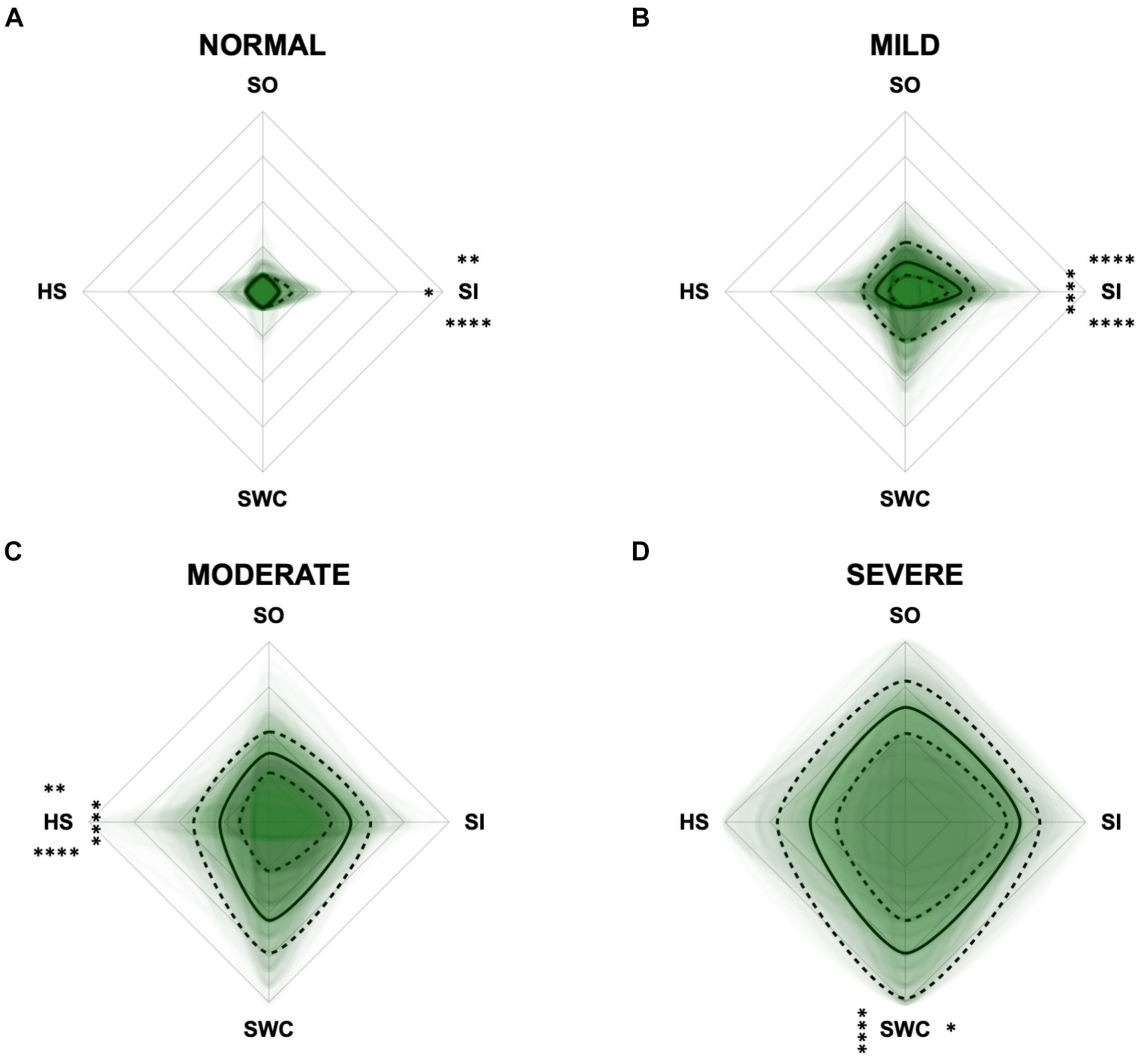
Radar plots consisting of all four CADES domains “spatial orientation” (SO), “social interaction” (SI), “sleep–wake-cycles” (SWC), and “house-soiling” (HS) within the different severity categories **(A)** normal, **(B)** mild, **(C)** moderate, and **(D)** severe. Each dog’s score pattern is represented by a transparent green area, and these areas overlap in each plot. The straight edges of the radar plot represent the maximum possible scoring of 100% per domain. The center of the plot is virtually set at −0.1 in order to highlight scores at zero more clearly. The solid black line inside the plots represents the median score in percent per domain in each plot; the inner and outer dotted lines represent the first and third quartile of the scores in percent per domain in each plot, respectively. Kruskal–Wallis tests (*p* ≤ 0.0001) followed by Dunn’s multiple comparisons tests were performed to compare each domain across all four severity categories (corresponding “normal” group as control category for each domain, *p* ≤ 0.0001, not illustrated in figure). Friedman tests (*p* ≤ 0.0001) followed by Dunn’s multiple comparisons tests were performed within each severity category to compare the scorings between different domains and assess their importance. Significant differences are illustrated by asterisks strategically placed around the domain names (*p ≤ 0.05, **p ≤ 0.01, and ****p ≤ 0.0001). The positions of the asterisks indicate the specific domain with which the comparison was conducted within the severity category. To illustrate, if an asterisk is positioned above a domain name, it signifies that the statistically significant comparison was made between that domain and the one positioned at the top of the entire plot.

To monitor the disease progression within the different domains of the CADES and CCAS, radar plots of the respective domain scores relative to the maximum possible score per domain were designed. For the CADES, the scores increased significantly in each domain along the progression (*p* ≤ 0.0001). However, the domains “spatial orientation,” “social interaction,” and “sleep–wake-cycles” were affected early during the progression from normal aging to mild/moderate, and severe impairment, whereas the “house-soiling” domain was affected later and to a lesser extent (Figure 7). More precisely, the “social interaction” domain developed before all other domains (Figures 7A,B), until the scorings of the “spatial orientation,” “sleep–wake-cycles,” and “social interaction” domains increased and adapted to each other (Figure 7C). In severely affected animals, the “spatial orientation” and “sleep–wake-cycles” domains finally became very relevant (Figure 7D).

**Figure 8:**
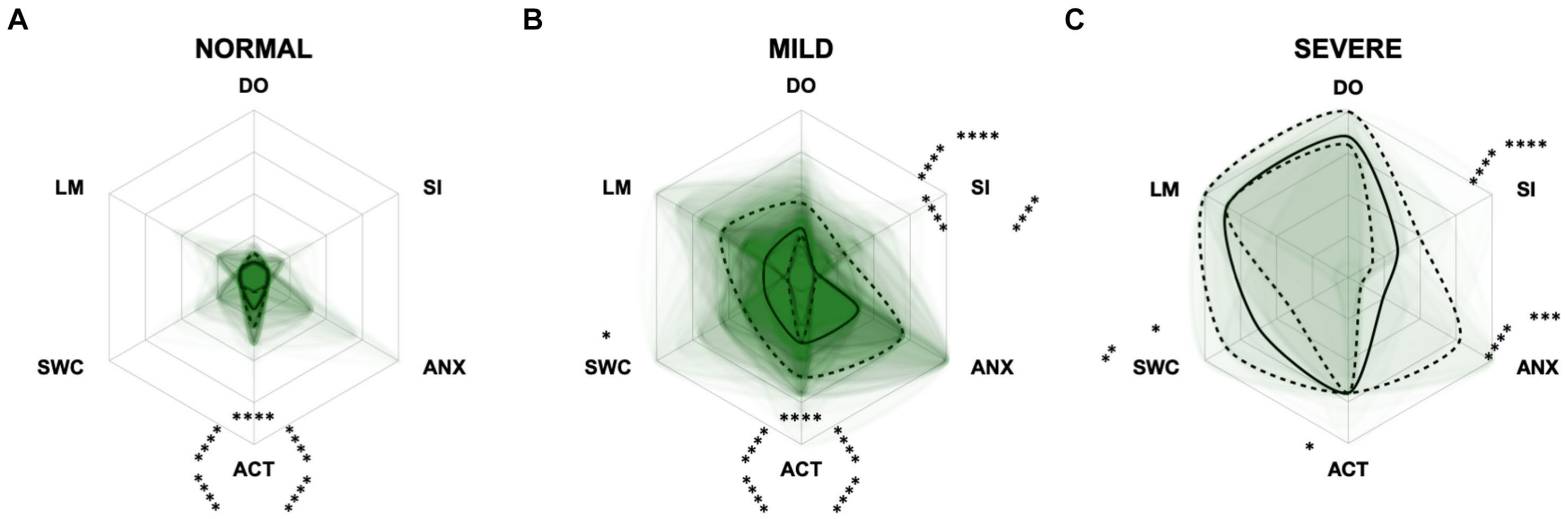
Radar plots consisting of all six CCAS domains “disorientation” (DO), “social interaction” (SI), “anxiety” (ANX), “activity level” (ACT), “sleep–wake-cycles” (SWC), and “learning and memory” (LM) within the different severity categories **(A)** normal, **(B)** mild, and **(C)** severe. Each dog’s score pattern is represented by a transparent green area, and these areas overlap in each plot. The straight edges of the radar plot represent the maximum possible scoring of 100% per domain. The center of the plot is virtually set at −0.1 in order to highlight scores at zero more clearly. The solid black line inside the plots represents the median score in percent per domain in each plot; the inner and outer dotted lines represent the first and third quartile of the scores in percent per domain in each plot, respectively. Kruskal–Wallis tests (*p* ≤ 0.0001) followed by Dunn’s multiple comparisons tests were performed to compare each domain across all three severity categories (corresponding “normal” group as control category for each domain, *p* ≤ 0.0001, not illustrated in figure). Friedman tests (*p* ≤ 0.0001) followed by Dunn’s multiple comparisons tests were performed within each severity category to compare the scorings between different domains and assess their importance. Significant differences are illustrated by asterisks strategically placed around the domain names (*p ≤ 0.05, **p ≤ 0.01, ***p ≤ 0.001, and ****p ≤ 0.0001). The positions of the asterisks indicate the specific domain with which the comparison was conducted within the severity category. To illustrate, if an asterisk is positioned above a domain name, it signifies that the statistically significant comparison was made between that domain and the one positioned at the top of the entire plot.

For the CCAS, the scores increased significantly in each domain along CCD progression (*p* ≤ 0.0001) compared to the “normal” group (Figure 8). Contrary to expectations, the scores in the “anxiety” domain showed an initial increase between the “normal” and “mild” category and then a slight decrease between the “mild” and “severe” category (Figures 8B,C). With increasing severity of CCD, the “activity level” domain in the CCAS was the first to show significant changes in comparison to other domains (Figures 8A,B). However, the “disorientation” and “learning, and memory” domains showed the highest margin of change among all domains as the disease progressed from normal to severe (Figure 8C). The domains “social interaction” and “anxiety” played a less important role along the entire CCD development in the CCAS questionnaire (Figures 8B,C), as underpinned by PCA (Figure 5B) and CFA (Figure 6B). Interestingly, the domain “social interaction” showed only little change in comparison to other domains along the severity categories, in contrast to the same domain in the CADES questionnaire.

#### 3.2.5 Evaluation of questionnaire results versus C-BARQ domains

**Figure 9:**
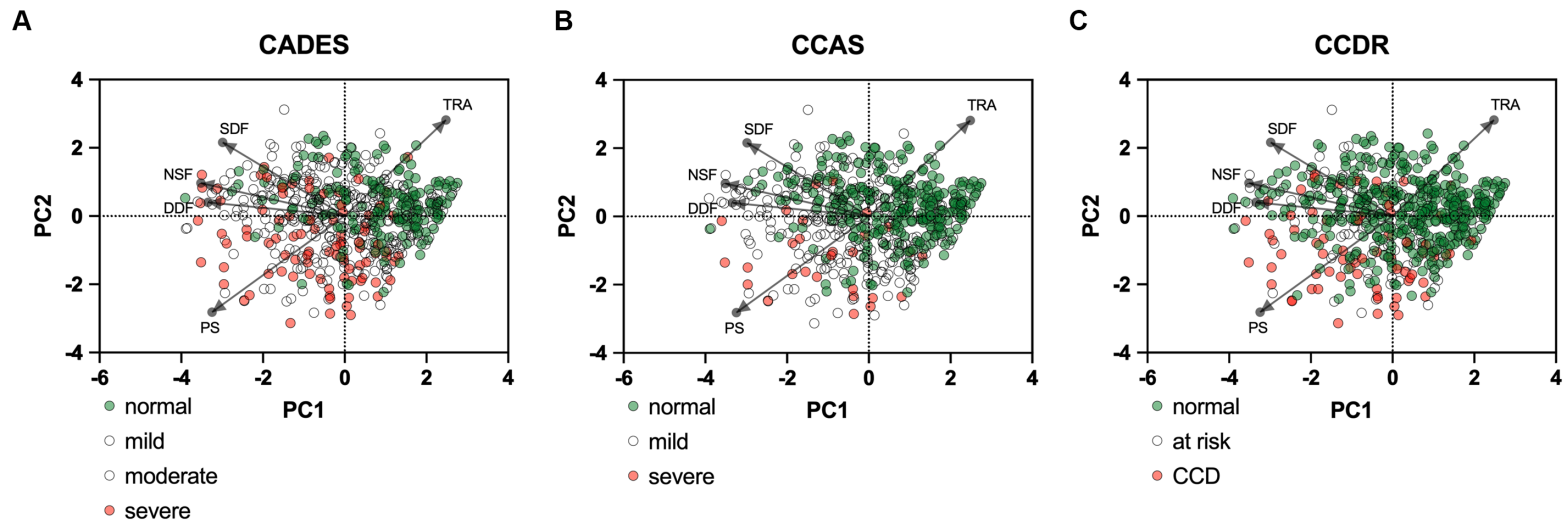
Principal component analysis (PCA) biplots of Canine cognitive dysfunction (CCD) scores and categorization in relation to the C-BARQ domains “dog-directed fear” (DDF), “stranger-directed fear” (SDF), “nonsocial fear” (NSF), “trainability” (TRA), and “pain sensitivity” (PS). The colored data points (PCA scores) represent each dog and the general severity progression patterns (from green to red), whereas the black arrows and points represent the loadings for each C-BARQ domain. Please note that dogs with mild/moderate signs or at risk for developing CCD are not displayed in color to increase visibility of the main progression patterns. **(A)** The progression pattern of CADES scores had a robust positive and negative relationship with the C-BARQ domains PS and TRA along CCD progression, respectively. **(B,C)** Similar relationships can be observed for the CCAS and CCDR, although the small number of severely affected dogs makes an interpretation challenging.

PCAs were performed to examine the relationship between the cognition assessing tools CADES, CCAS, and CCDR scoring and a general behavioral assessment tool, i.e., the C-BARQ questionnaire, which was used here to investigate aspects of fear, trainability, and pain sensitivity (Figure 9). Overall, there was a negative correlation of the C-BARQ domains “trainability” and “pain sensitivity.” These also had the largest influence on the CADES, CCAS, and CCDR severity categories (no, mild, moderate, severe impairment). Other domains, such as “nonsocial fear” and “dog-directed fear,” showed less association with the main progression patterns of CCD, while the domain “stranger-directed fear” appeared to be the least important.

**Figure 10:**
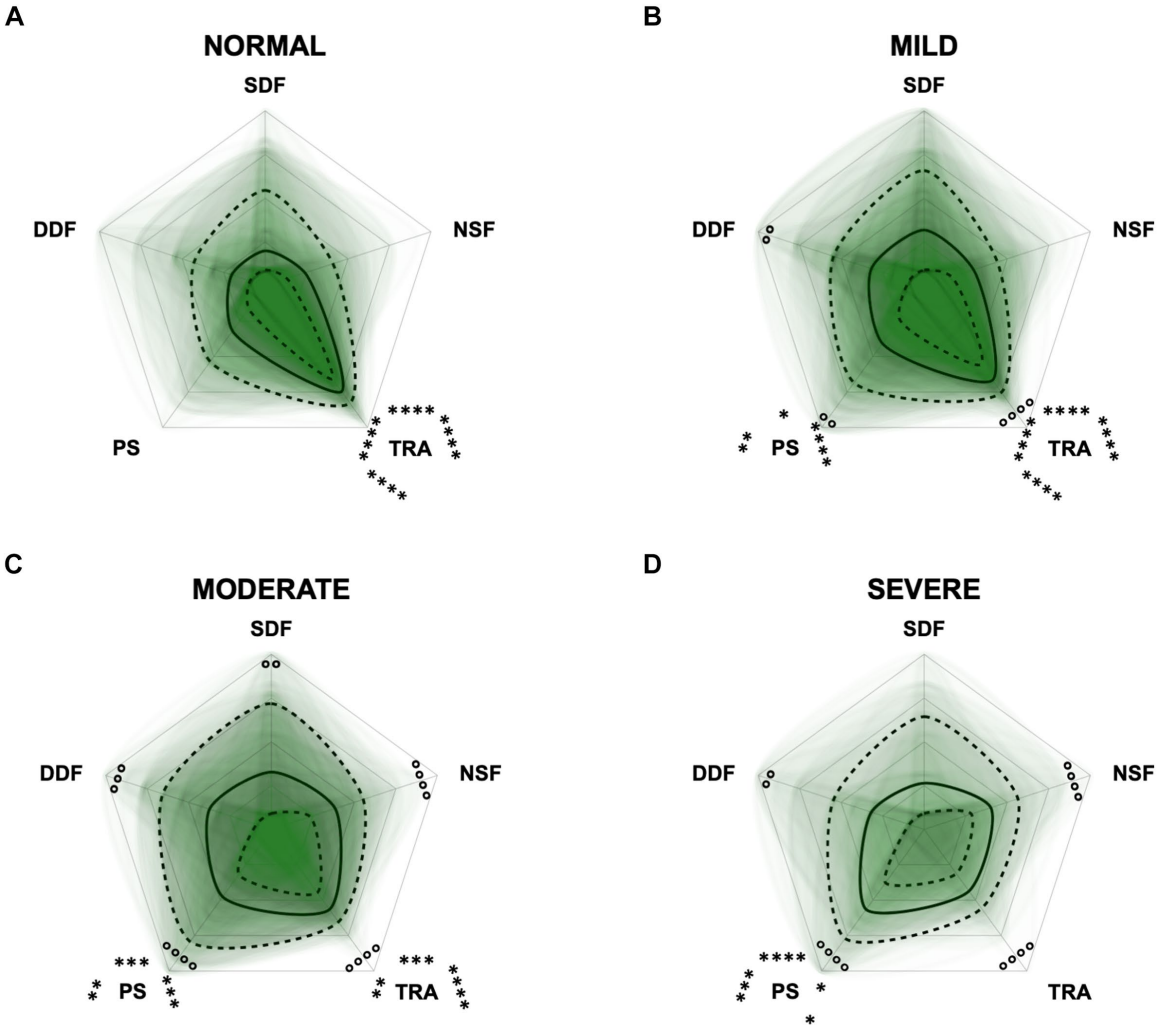
Radar plots consisting of the five C-BARQ domains “dog-directed fear” (DDF), “stranger-directed fear” (SDF), “nonsocial fear” (NSF), “trainability” (TRA), and “pain sensitivity” (PS) within the different severity categories **(A)** normal, **(B)** mild, **(C)** moderate, and **(D)** severe of the CADES questionnaire. Other behavior domains of the C-BARQ, such as “chasing” and “excitability,” were excluded as they seemed less relevant to the diagnosis of Canine cognitive dysfunction. Each dog’s score pattern is represented by a transparent green area, and these areas overlap in each plot. The straight edges of the radar plot represent the maximum possible scoring of 100% per domain. The center of the plot is virtually set at −0.1 in order to highlight scores at zero more clearly. The solid black line inside the plots represents the median score in percent per domain in each plot; the inner and outer dotted lines represent the first and third quartile of the scores in percent per domain in each plot, respectively. Kruskal–Wallis tests (*p* ≤ 0.05) followed by Dunn’s multiple comparisons tests were performed to compare each domain across all four severity categories (corresponding “normal” group as control category for each domain; significant differences illustrated by circles at each domain; *p* ≤ 0.01, *p* ≤ 0.001, and *p* ≤ 0.0001). Friedman tests (*p* ≤ 0.0001) followed by Dunn’s multiple comparisons tests were performed within each severity category to compare the scorings between different domains and assess their importance. Significant differences are illustrated by asterisks strategically placed around the domain names (*p ≤ 0.05, **p ≤ 0.01, ***p ≤ 0.001, and ****p ≤ 0.0001). The positions of the asterisks indicate the specific domain with which the comparison was conducted within the severity category. To illustrate, if an asterisk is positioned above a domain name, it signifies that the statistically significant comparison was made between that domain and the one positioned at the top of the entire plot.

**Figure 11:**
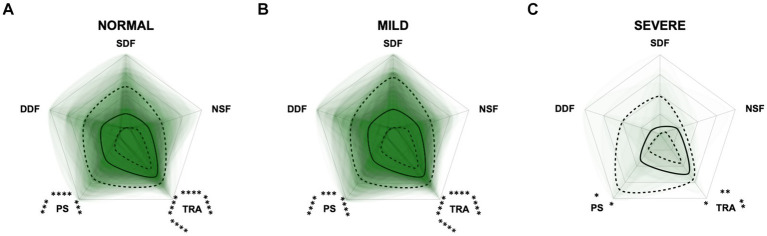
Radar plots consisting of the five C-BARQ domains “dog-directed fear” (DDF), “stranger-directed fear” (SDF), “nonsocial fear” (NSF), “trainability” (TRA), and “pain sensitivity” (PS) within the different severity categories **(A)** normal, **(B)** mild, and **(C)** severe of the CCAS questionnaire. Other behavior domains of the C-BARQ, such as “chasing” and “excitability,” were excluded as they seemed less relevant to the diagnosis of Canine cognitive dysfunction. Each dog’s score pattern is represented by a transparent green area, and these areas overlap in each plot. The straight edges of the radar plot represent the maximum possible scoring of 100% per domain. The center of the plot is virtually set at −0.1 in order to highlight scores at zero more clearly. The solid black line inside the plots represents the median score in percent per domain in each plot; the inner and outer dotted lines represent the first and third quartile of the scores in percent per domain in each plot, respectively. Kruskal–Wallis tests followed by Dunn’s multiple comparisons tests were performed to compare each domain across all three severity categories (corresponding “normal” group as control category for each domain; not significant). Friedman tests (*p* ≤ 0.0001) followed by Dunn’s multiple comparisons tests were performed within each severity category to compare the scorings between different domains and assess their importance. Significant differences are illustrated by asterisks strategically placed around the domain names (*p ≤ 0.05, **p ≤ 0.01, ***p ≤ 0.001, and ****p ≤ 0.0001). The positions of the asterisks indicate the specific domain with which the comparison was conducted within the severity category. To illustrate, if an asterisk is positioned above a domain name, it signifies that the statistically significant comparison was made between that domain and the one positioned at the top of the entire plot.

**Figure 12:**
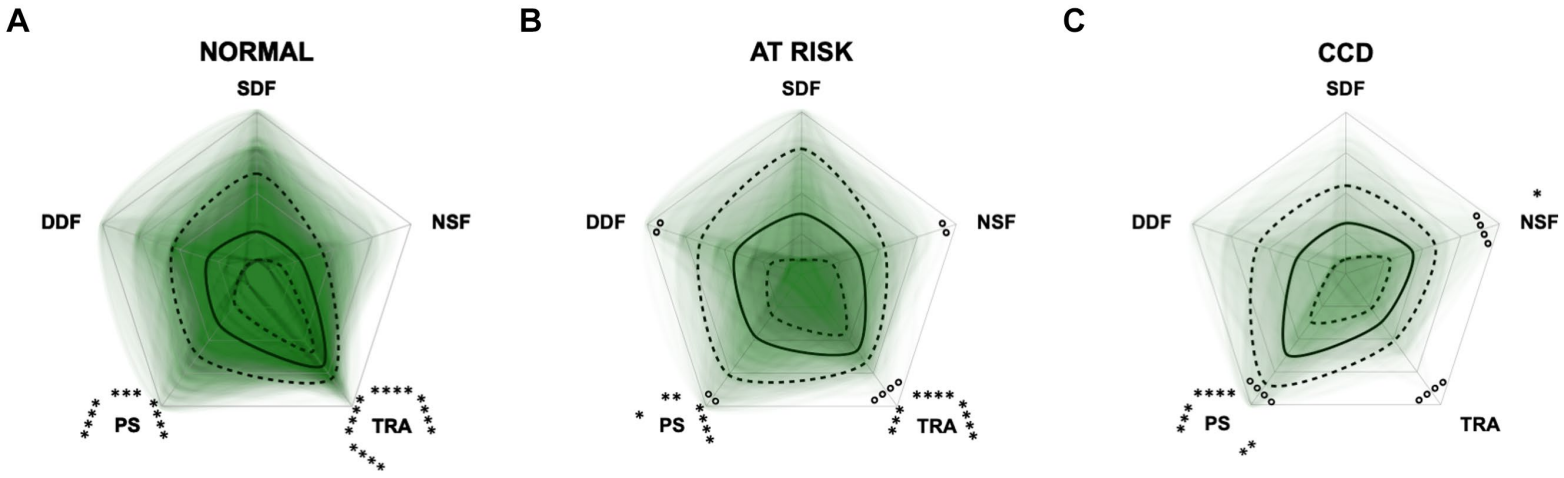
Radar plots consisting of the five C-BARQ domains “dog-directed fear” (DDF), “stranger-directed fear” (SDF), “nonsocial fear” (NSF), “trainability” (TRA), and “pain sensitivity” (PS) within the different severity categories **(A)** normal, **(B)** at risk, and **(C)** Canine cognitive dysfunction (CCD) of the CCDR questionnaire. Other behavior domains of the C-BARQ, such as “chasing” and “excitability,” were excluded as they seemed less relevant to the diagnosis of CCD. Each dog’s score pattern is represented by a transparent green area, and these areas overlap in each plot. The straight edges of the radar plot represent the maximum possible scoring of 100% per domain. The center of the plot is virtually set at −0.1 in order to highlight scores at zero more clearly. The solid black line inside the plots represents the median score in percent per domain in each plot; the inner and outer dotted lines represent the first and third quartile of the scores in percent per domain in each plot, respectively. Kruskal–Wallis tests (*p* ≤ 0.01) followed by Dunn’s multiple comparisons tests were performed to compare each domain across all three severity categories (corresponding “normal” group as control category for each domain; significant differences illustrated by circles at each domain; *p* ≤ 0.01 and *p* ≤ 0.0001). Friedman tests (*p* ≤ 0.0001) followed by Dunn’s multiple comparisons tests were performed within each severity category to compare the scorings between different domains and assess their importance. Significant differences are illustrated by asterisks strategically placed around the domain names (*p ≤ 0.05, **p ≤ 0.01, ***p ≤ 0.001, and ****p ≤ 0.0001). The positions of the asterisks indicate the specific domain with which the comparison was conducted within the severity category. To illustrate, if an asterisk is positioned above a domain name, it signifies that the statistically significant comparison was made between that domain and the one positioned at the top of the entire plot.

Additional radar plots corroborate the observed association between higher test scores and C-BARQ behavior domains. These showed as well that higher CCD questionnaire scores are associated with lower trainability and increased pain sensitivity and nonsocial fear to some extent. Questionnaire scores appeared to have a smaller association with the C-BARQ domain “dog-directed fear,” while the domain “stranger-directed fear” was not significantly related to CCD severity across the score systems (Figures 10–12). More precisely, the CADES showed a clear decrease in the domain “trainability” and an increase in the domain “pain sensitivity” along the progression of CCD, and also in the relative importance of domains in each severity category. While the “trainability” domain showed the highest scores in the “normal” group, this relationship switched to a more pronounced “pain sensitivity” domain in the group with severely affected animals. The domains of “nonsocial fear” and, to a lesser extent, “dog-directed fear” increased along CCD progression, as well (Figure 10). In the CCAS assessment, these relationships were less pronounced. There were no significant changes in domain scoring along CCD progression, and the domains of “trainability” and “pain sensitivity” have aligned to some extent (Figure 11). Similarly to the CADES, there was an observable increase in the domains of “pain sensitivity” and “nonsocial fear” in the CCDR, whereas scoring in the domain “trainability” decreased along CCD progression (Figure 12). Interestingly, scores in social fear-associated C-BARQ domains increased especially in mildly affected animals throughout the questionnaires compared to the corresponding “normal” groups, while scores were unchanged or only slightly changed in the “severe” groups compared to the corresponding “normal” groups. The CADES and CCDR systems exhibited these effects most prominently.

In the severely affected groups, the domain “nonsocial fear” showed significantly lower behavioral scores (i.e., decreased nonsocial fear) in the CCAS compared to the other questionnaires, respectively (*p* ≤ 0.01). The scores of the domain “trainability” were significantly increased (i.e., increased trainability) in the severely affected groups assessed with the CCAS compared to the CADES (*p* = 0.0013) and to the CCDR (*p* ≤ 0.0001). However, there were no significant differences in the domain “pain sensitivity” across questionnaires in the groups with severely affected animals.

## 4 Discussion

The differences between the three chosen screening questionnaires have not been investigated to date and their ability to assess CCD has not yet been compared. Even though their classifications into different stages of CCD differed between the three questionnaires, the present study found a significant correlation between them, which was underpinned by explorative PCAs and construct concordance. The strong agreement between the CADES and the CCAS is plausible, as the questionnaires include similar items, such as questions from the domains “spatial orientation” or “disorientation,” “social interaction,” and “sleep–wake-cycles.” They also both identify similar behavior frequencies, e.g., “never,” “once in the last six months,” “once a month,” “once a week,” and “every day.” More specifically, alterations of social interaction (CADES) and activity (CCAS) may serve as early predictors of CCD, while limitations in spatial orientation generally clearly emerge as key features in advanced cases. There was a weaker agreement between both questionnaires and the CCDR, as it does not differentiate between different domains, but also rates frequencies of behavioral entities like the other two questionnaires. However, looking at the questionnaires from a different perspective—such as using behavioral elements of the scientifically popular C-BARQ as a template and observing the classification into dementia severity categories—revealed some discrepancies in this respect. For instance, this becames apparent in the C-BARQ domains of “trainability” and “pain sensitivity,” which seem to play a more important and dynamic role in the CADES and CCDR systems than in the CCAS system, respectively. In general, scores in fear and anxiety domains were mainly increased in animals with mild CCD while they were decreased in animals with severe CCD.

The CADES classified more dogs as showing mild to moderate cognitive impairment than the other two questionnaires, which identified more dogs as aging normally. As the lower score cut-off of nearly 40% of the total score range for the CCDR is much higher than the cut-offs of the CADES and the CCAS, which are 7 and 10%, respectively, this might explain the smaller number of mildly or severely affected dogs on the CCDR scale (Figure 1). As previously reported by Salvin et al. (25) and Schütt et al. (28), the CCDR is especially suitable for detecting severe signs of CCD.

Within the group classified as “normal” by the CCDR, there were two clusters of dogs: Those with scores of around 35 (33% of the total scale) and those with scores of around 20 (10% of the total scale) (Figure 1C). When filling in the questionnaire, changes in frequency of behavioral entities over the last six months are assessed and scored from one (much less) to five (much more) in the second half of the questionnaire (six questions). If the frequency did not change, this would give the dog three points per question. Scores of two of the five questions are multiplied or tripled. Thus, obtaining a score lower than 30 for a patient that has never shown signs of cognitive impairment is unlikely, given the wording of the second half of questions. To correctly answer the questions “compared to 6 months ago, how often does your dog now…,” an owner would respond with “the same” if their dog did not exhibit these behavioral changes in the past and still does not. This, however, will give the dog three, or even six or nine points, leading to a higher total score. A completely unaffected dog would therefore probably reach a score of 30–36. The cluster of lower scores in this study may either be due to a drastic improvement of behavioral changes over the last 6 months but could also reflect lack of focus by responders. Especially if their dogs are not exhibiting signs of cognitive impairment, owners may be less inclined to read questions carefully. Changing the cut-offs could be useful to improve the CCDR’s ability to detect mildly affected dogs, but further studies are required to determine the reliability and validity of such adaptions.

Even though the frequency assessment and severity category cut-offs of the CADES and CCAS were similar, the CADES still identified fewer normally aging (29% vs. 46.5%), but also more severely affected (15% vs. 4.5%) dogs than the CCAS, respectively. The latter concurs with findings from Le Brech et al. (27), who reported in their 2022 study that the CCAS identified very few severely affected dogs and hypothesized that this was due to very poor general health and consequent exclusion from their study. As the present study was based on an owner questionnaire, no such exclusion criteria were applied, but the results were similar. Therefore, the CCAS may not be ideal for detection of severe cognitive impairment. Further studies could investigate different cut-offs and improve the questionnaires’ ability to identify severely affected CCD patients.

Group comparisons and the REML model indicated that increasing age had an impairing effect on canine cognitive function, in general. As other studies have shown similar results (1, 2, 50), this was not surprising. However, the analyses showed that there was a large fraction of dogs in the “normal” categories of the questionnaires that exhibited healthy aging into old age without any impact on CCD-score development. On the other hand, aging demonstrated an increased impact on CCD scores when dogs were in the mildly or severely CCD-affected groups, which was well observed in the CADES and CCAS systems. This observation emphasizes that age could serve as a progressive factor in CCD when there are already cognitive impairments in dogs, and that the CADES and CCAS can be appropriate tools for distinguishing between healthy and diseased aging.

We were also interested in a more detailed examination of the domains of each questionnaire to investigate the influence these may have on the total scores. Therefore, we performed PCAs and CFAs with the CADES and CCAS domains. Furthermore, radar plots for the corresponding domains serve as an illustration of how the individual domains perform within the severity categories. Thereby they provide an impression of how the domains can change along the progression of CCD and how important their contribution to the general progression of CCD is. For the CADES, we found that the domains “spatial orientation,” “social interaction,” and “sleep–wake-cycles” were most important, whereas “house-soiling” was less relevant and correlated less with those other domains. Surprisingly, this was different to the findings of Madari et al. (26), who performed PCA in their study and found that the scores in the “sleep–wake-cycles” domain correlated less with the scores of the other domains and was generally less important for the general performance of the CADES. For the CCAS, the domains “disorientation,” “activity level,” “sleep–wake-cycles,” and “learning and memory” were most important. The domains “social interaction” and “anxiety,” in contrast to the CADES, appeared to be less relevant and correlated less with the other domains. A detailed examination of the “social interaction” domain could explain this discrepancy, as the CADES scans this domain with a more nuanced approach compared to the CCAS. The limited impact of the “anxiety” domain on the overall performance of the CCAS may be attributed to a significant asymmetry in the score distribution (Supplementary Table S2). This domain contributes a maximum of 3 points to the overall score out of a possible 69 points. This rough point allocation could account for the poor contribution. However, to obtain a more detailed view of individual domains along CCD progression, we examined them within each severity category using radar plots.

The radar plots revealed that the CADES domain “social interaction” developed early on, demonstrating its early emergence or advancement in comparison to other domains. It was the domain with the highest scores in the groups “normal” and “mild cognitive impairment,” which concurs with the findings of Madari et al. (26). In the moderately and severely affected groups, all four domains became more relevant, with the domain “house-soiling” showing the least development along CCD progression (Figure 7). Since CCD is a neurodegenerative disorder (15), behavioral changes may be subtle and affect only a few cognitive domains in the beginning. As the disease progresses, it is more likely that more domains become affected and the severity of behavioral changes increases (8, 26) and, therefore, test scores in all domains increase. As stated above, the score ranges in the CCAS domains were unevenly distributed. In order to enable better comparability in the radar plots, domains in each questionnaire were analyzed as relative values with the maximum possible score per domain as 100% reference. Along CCD progression, the CCAS domain “activity level” developed early on and the other domains followed in the “mild” and “severe” groups. However, the development of the domains “social interaction” and “anxiety” stagnated to a certain extent and played a less relevant role in severe cases which, again, might be due to the aspects explained in the preceding section (Figure 8). Interestingly, the mildly affected animals exhibited increased “anxiety” values, which were decreased again in the “severe” group. This may indicate that after an initial increase in anxiety during CCD development, it subsequently plays a subordinate role in severe cases due to a decline in cognition and the ability to assess situations properly. However, it should also be noted that the “severe” group in the CCAS is small. In general, alterations of social interaction (CADES) and activity (CCAS) may serve as early indicators of CCD, while impairments in spatial orientation typically emerge as key features in advanced CCD cases. Fear and anxiety appears to increase in mildly affected animals and then decrease in severe CCD cases.

To integrate additional behavioral entities not covered by the CCD questionnaires, C-BARQ questionnaires were completed for each dog (48). In this way, a potential grade of sensitivity of the different dementia scales towards some domains of the prominently used C-BARQ was investigated. The performance of C-BARQ domains was then elucidated using PCA and radar plots for each CCD questionnaire as the groups progressed from normal to severe. The C-BARQ was developed to assess dog behavior and consists of questions regarding fear, aggression, separation anxiety, attachment, trainability, chasing, excitability and pain sensitivity. We used domains of the C-BARQ that might be affected by CCD, assessing fear and anxiety, trainability, and pain sensitivity. Especially, the domains “nonsocial fear,” “trainability,” and “pain sensitivity” played a role in the CCD progression dynamics as well as within the different severity categories. While the CADES and the CCDR showed a clear shift between decreased pain sensitivity (low scores) and increased trainability (high scores) in healthy animals to increased pain sensitivity and decreased trainability in severely CCD-affected animals (Figures 10, 12), these alterations were less pronounced in the CCAS questionnaire. In the latter, there is a tendency for approximation between domain scores rather than a substantial shift in the relation of these scores (Figure 11). Additionally, there are no significant differences in the C-BARQ domains between the severity categories of the CCAS.

In general, higher CCD test scores also correlated with higher levels of the C-BARQ domains assessing fear. While “nonsocial fear” increased along CCD progression, the domains “dog-directed fear” and “stranger-directed fear” only showed increased values in mildly affected animals and decreased in the severely affected groups. This development could be observed more effectively in the CADES and CCDR questionnaires than in the CCAS. It reinforces the observation, as noted in the CCAS domain of “anxiety,” that in mild/moderate CCD the scores of the socially associated fear domains of the C-BARQ appear to increase, but then decrease to normal levels in the severely affected groups. In one study (26), it was found that fear and anxiety did not correlate with the severity of CCD and scores in this domain were similar between cognitively normal and impaired dogs. This was therefore only partially true in the present study.

The CADES and the CCDR appear to intercept and resolve certain dynamics in behavioral domains more reliably than the CCAS, at least in the context of the C-BARQ. This represents a discrepancy with the strong agreement between individual CADES and CCAS scores (Table 1 and Figure 4). Variations in the thresholds of the severity classification and their reliability may play a decisive role here as they exhibit significant distinctions between questionnaires. This highlights how sensitive the classification is for the clinical outcome. Further studies are needed to investigate reliability and quality of classification thresholds of CCD questionnaires in terms of diagnostic outcomes.

Lastly, this study confirms that CCD is still a highly underdiagnosed condition, as Salvin et al. (18) have previously reported. The diagnosis rate in our study was 15%, even though, depending on the screening questionnaire, at least one-third of the dogs were showing signs of cognitive decline. Reasons for this could be a persisting lack of awareness of the disease, as well as refusal or inability to recognize the observed behavioral changes as symptoms of CCD. It is possible that owners believe the changes their dog is displaying to be part of the normal aging process and that treatment of this is impossible (31).

This study has multiple limitations, the most relevant one being that the owners were not interviewed individually and so the reported behavioral changes and their severity may be influenced by the subjective perception of the owners. Since we did not have access to clinical records and did not evaluate the patients ourselves, we cannot exclude patients with a false positive or false negative assessment made by one of the three screening questionnaires. Furthermore, this was an online questionnaire and the people who responded may not be representative for the overall population, since the study population was biased toward female owners between the ages of 46–60. In addition, different health conditions and medications (especially for brain health) might have had a potential direct impact on the outcomes of the analyses. However, subgroup analyses were not in the scope of this study.

It is important that veterinarians keep the differences between screening questionnaires in mind when diagnosing dogs with CCD, as this can have a big impact on diagnosis and treatment plan. The results of this study may be helpful for future studies that aim to explore methods of diagnosing CCD.

## 5 Conclusion

The scores of the investigated questionnaires correlated well with each other, especially those of the CADES and CCAS, and all of them may be useful for diagnosing CCD. The CADES classified dogs into more differentiated severity groups, thus potentially giving a more realistic and “predictive” evaluation of CCD. Questionnaire domains such as “spatial orientation” or “disorientation,” “social interaction,” “sleep–wake-cycles,” “learning and memory” as well as “activity level” seem to play important roles in the overall CADES and CCAS questionnaire performances, whereas fear and anxiety seem to be more pronounced in mildly affected animals but not in severely affected animals. The domains “social interaction” (CADES) and “activity level” (CCAS), in particular, exhibited changes early on along CCD progression, whereas domains such as “spatial orientation” or “disorientation” served as solid markers for the severe forms of CCD. The CCDR works particularly well for dogs with severe signs of CCD. However, this study also highlights the differences in structures and results of the scales, which can have an influence on the final diagnosis. For instance, the CADES and CCDR showed sensitive performance within the framework of established behavior assessment tools such as the C-BARQ (especially for the domains “trainability,” “pain sensitivity,” and “nonsocial fear”), while the CCAS showed inconclusive results along progression in this respect. These discrepancies highlight the issue of severity category classification which varies significantly between the investigated score systems. As described here, the comprehensive analyses of the three dementia questionnaires in the current study might elucidate which internal, external, or translational factors of the scales may play a role in deciding what questionnaire might be suitable for a specific patient. Both validation studies for a better-resolved classification of the diagnosis as well as longitudinal studies may be useful to determine further which questionnaire is best suited for the early and reliable detection of CCD.

## Data availability statement

The raw data supporting the conclusions of this article will be made available by the authors, without undue reservation.

## Ethics statement

Ethical approval was not required for the studies involving animals in accordance with the local legislation and institutional requirements because this study sampled only filled in questionnaires assessing observational information from privately owned dogs. There were no harmful animal experiments carried out and no damage, harm, or pain was provoked in animals. Written informed consent was obtained from the owners for the participation of their animals in this study.

## Author contributions

JH: Conceptualization, Data curation, Formal analysis, Investigation, Methodology, Resources, Software, Validation, Visualization, Writing – original draft, Writing – review & editing. SM: Conceptualization, Data curation, Formal analysis, Investigation, Methodology, Project administration, Software, Supervision, Validation, Visualization, Writing – original draft, Writing – review & editing, Resources. NM: Conceptualization, Methodology, Project administration, Validation, Writing – review & editing. FT: Writing – review & editing. MC: Writing – review & editing. ST: Data curation, Formal analysis, Funding acquisition, Methodology, Software, Validation, Visualization, Writing – review & editing. HV: Conceptualization, Funding acquisition, Methodology, Project administration, Resources, Supervision, Validation, Writing – review & editing, Visualization.
