# Supplementary material for: Comparing standard screening questionnaires of canine behavior for assessment of cognitive dysfunction
Source: Front Vet Sci. 2024 May 21;11:1374511. doi: 10.3389/fvets.2024.1374511 (PMC11149356; doi:10.3389/fvets.2024.1374511)
Supplement: Supplementary file 1 [file Data_Sheet_1.DOCX]

Supplementary Material

**Supplementary Table 1** Score range per CADES domain and the overall total

|  | Min | Max |
| --- | --- | --- |
| Spatial orientation | 0 | 25 |
| Social interaction | 0 | 25 |
| Sleep-wake-cycles | 0 | 20 |
| House-soiling | 0 | 25 |
| Total | 0 | 95 |

**Supplementary Table 2** Score range per CCAS domain and the overall total

|  | Min | Max |
| --- | --- | --- |
| Disorientation | 0 | 36 |
| Social interaction | 0 | 9 |
| Sleep-wake-cycles | 0 | 6 |
| Learning and Memory | 0 | 6 |
| Activity Level | 0 | 9 |
| Anxiety | 0 | 3 |
| Total | 0 | 69 |

**Supplementary Table 3** Administered medications to dogs during the study

| **Medications** | **n and % of dogs receiving medication** |
| --- | --- |
| Brain medications, among others: | n = 116/597, 19% |
| - Propentofylline | n = 85/597, 14% |
| - Selegiline | n = 8/597, 1% |
| Other medications/treatments, among others: | n = 269/597, 45% |
| - Routine medications (e.g., deworming, vaccination) | n = 255/597, 43% |
| - Antibiotics | n = 81/597, 14% |
| - Hormonal medications | n = 62/597, 10% |
| - Dietary interventions | n = 20/597, 3% |
| - Pain medications | n = 19/597, 3% |
| - Sympathomimetics | n = 18/597, 3% |
| - Immunosuppressants | n = 10/597, 2% |
| - Heart medications | n = 3/597, 1% |
| No medications | n = 147/597, 25% |


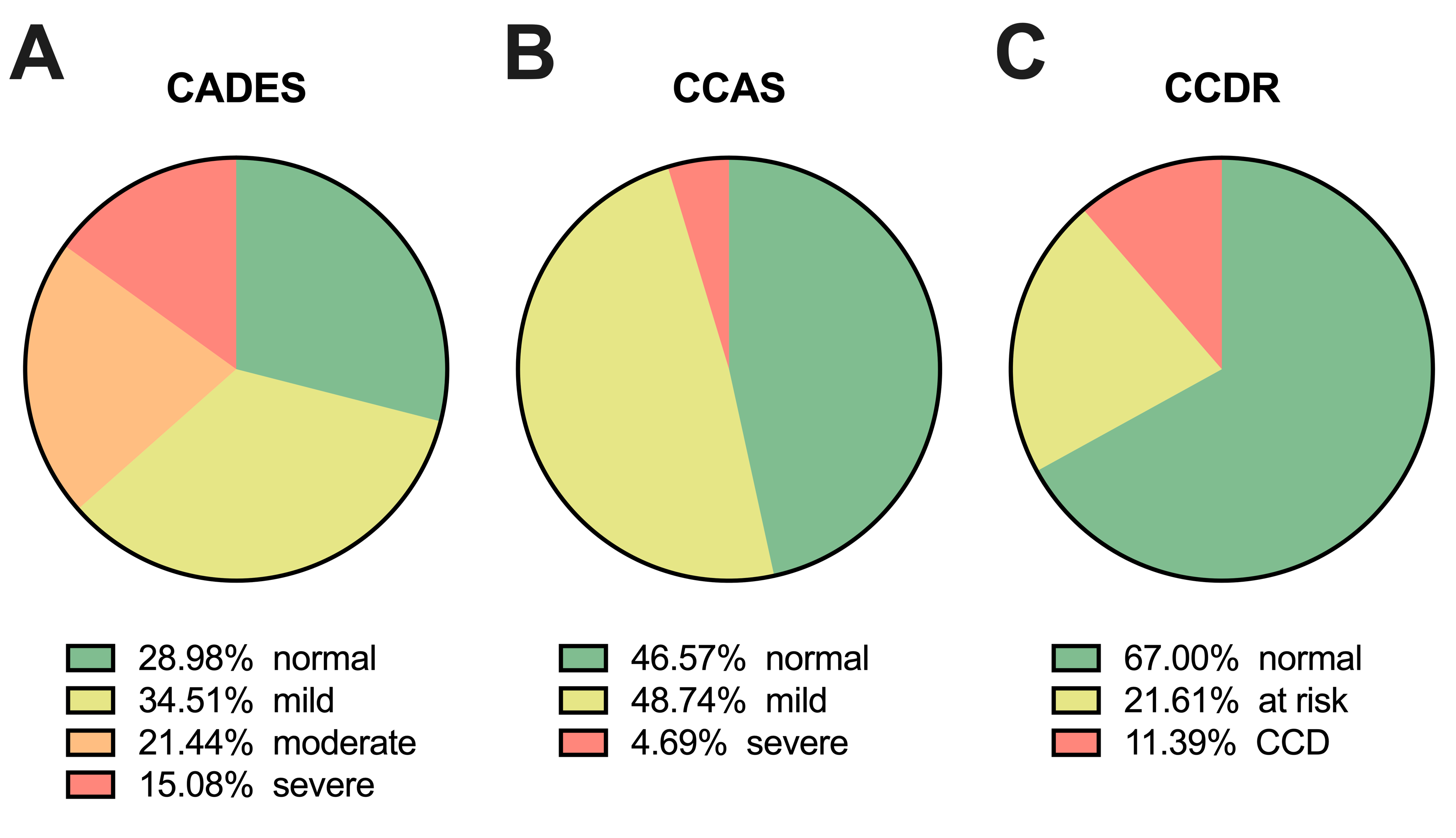


**Supplementary Figure 1** Distribution of scores of all dogs (n = 597) within different categories among the Canine cognitive dysfunction (CCD) questionnaires (A) CADES, (B) CCAS, and (C) CCDR.


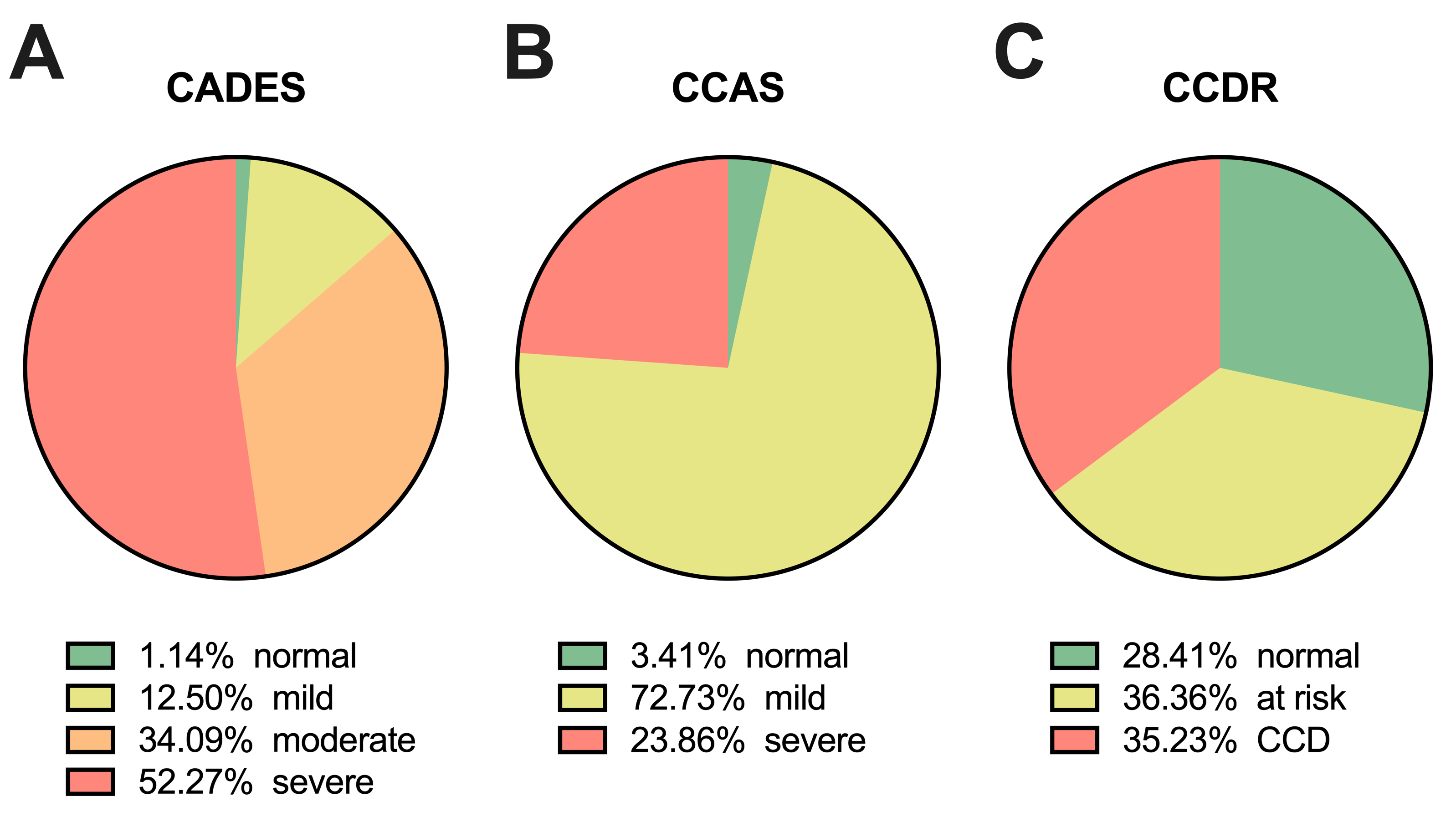


**Supplementary Figure 2** Distributions of scores of dogs previously diagnosed with canine cognitive dysfunction (CCD, n = 88) by a veterinarian within different categories among the CCD questionnaires (A) CADES, (B) CCAS, and (C) CCDR.
